# Practices of research data curation in institutional repositories: A qualitative view from repository staff

**DOI:** 10.1371/journal.pone.0173987

**Published:** 2017-03-16

**Authors:** Dong Joon Lee, Besiki Stvilia

**Affiliations:** 1 University Libraries, Texas A&M University, College Station, Texas, United States of America; 2 School of Information, Florida State University, Tallahassee, Florida, United States of America; University of Cape Town, SOUTH AFRICA

## Abstract

The importance of managing research data has been emphasized by the government, funding agencies, and scholarly communities. Increased access to research data increases the impact and efficiency of scientific activities and funding. Thus, many research institutions have established or plan to establish research data curation services as part of their Institutional Repositories (IRs). However, in order to design effective research data curation services in IRs, and to build active research data providers and user communities around those IRs, it is essential to study current data curation practices and provide rich descriptions of the sociotechnical factors and relationships shaping those practices. Based on 13 interviews with 15 IR staff members from 13 large research universities in the United States, this paper provides a rich, qualitative description of research data curation and use practices in IRs. In particular, the paper identifies data curation and use activities in IRs, as well as their structures, roles played, skills needed, contradictions and problems present, solutions sought, and workarounds applied. The paper can inform the development of best practice guides, infrastructure and service templates, as well as education in research data curation in Library and Information Science (LIS) schools.

## Introduction

The access and sharing of research data have been emphasized by the government [1], funding agencies [2–4] and scholarly communities [5,6]. The increased access to research data elevates the impact, efficiency, and effectiveness of scientific activities and funding opportunities. The access, however, is facilitated not just by appropriate policies, but also by the employment of effective infrastructure mechanisms, including enhancing data with effective metadata [7]. There is an increasing number of academic institutions that plan to provide research data services through their Institutional Repositories (IRs; [8,9]). Although many research universities already have operational IRs that provide open access to the digital content produced by the universities’ communities, only a small number of institutions provide research data services through their IRs [10].

There are three types of repositories: domain, discipline and institutional [11]. The main difference among them is the scope of content they collect and manage. IRs can be defined as “a set of services that an institution provides to the members of its community for the management and dissemination of digital materials created by the institution and its community members” [12]. IRs offer various benefits to support curatorial activities, including preserving, discovering, controlling, reusing and repurposing institutional intellectual content [13,14]. In addition, IRs provide channels for content dissemination and communication, which may increase an institution’s name value by aggregating and showcasing intellectual work produced by its communities [11,12,14]. The potential increase in an institution’s name value and the emphasis that major funding agencies place on research data sharing [2,3,15] motivates many institutions to establish IRs and related value added services for research data [8,16]. The literature shows that IRs storing and curating research data can increase its value, credibility, and reuse [11,17].

Having a good understanding of the current research data curation practices in IRs is essential for providing effective data curation services and achieving the objectives of IRs, which include but are not limited to sharing, accessing, controlling and preserving knowledge and data [13,18]. In particular, detailed descriptions of research data management related activities, user activities and needs, activity structures, roles played, and tools used can inform the development of best practice guides and infrastructure templates for IRs [19]. Those knowledge tools then could be used by the institutions currently implementing institutional data repositories and/or training IR curators.

Metadata management is one of the essential components of research data curation. Furthermore, Linked Data technologies are increasingly used to expose, discover, link and integrate knowledge, metadata, and data curated by libraries and IRs [20,21]. Consequently, it also becomes increasingly important to understand and coordinate identifier metadata (the essential component of any Resource Description Framework (RDF) based serialization and therefore any Linked Data implementation), as well as to ensure the quality and reusability of those identifiers. For example, the identifiers (e.g., Open Researcher and Contributor ID [ORCID], Archival Resource Key [ARK]) that can be assigned to various entities (e.g., person and event) and related identity profiles currently have significant commercial and community input [22]. There is a significant body of research on metadata quality and reusability in general [23,24], however, there is a dearth of research, on identifier metadata quality, uses and practices for research data in the context of IRs.

## Problem statement and research questions

The purpose of this descriptive study is to examine research data curation practices in IRs. Although there have been many previous studies of IRs [11,12,13,16], to the best of our knowledge, there has not been an in-depth, qualitative study to provide rich, detailed descriptions of data curation practices. This study addresses that need by examining data management and use activities in IRs, including the roles played, tools used, conventions and rules followed, contradictions among different components of the activities, and solutions sought to address those contradictions. Findings of the study can inform the design and planning of data curation services in IRs. The study’s findings also can guide IR curators’ training in LIS schools and professional organizations. The study examined the following research questions:

RQ 1. What are some of the data curation/management activities in IRs?
RQ 2. What are some of the user activities in IRs?
RQ 3. What are the structures of those activities (i.e., objectives, goals, division of labor, data types, tools, policies, rules, norms and contradictions)?

## Literature review

Three types of repositories (i.e., domain, discipline and institutional) exist [11]. The main difference between them is the granularity of the organizations that operate the repositories. For example, a chemistry community may develop a domain repository; a crystallography community may operate a disciplinary repository; and a university may run an institutional repository (IR). An IR may increase an institution’s name value by providing access to intellectual work produced by its communities [11,12,14]. This access, along with an emphasis on research data archiving and sharing from major funding agencies [2,3,15], motivates many institutions to put their efforts into the development of IRs and services for research data [8,16]. According to many researchers, IRs storing and curating research data can help reuse and repurpose the data [17] and increase the value and credibility of the data [11].

Curation of research data is the process of managing research data throughout its lifecycle for long-term availability and reusability [25–27]. Curation and its activities facilitate discovery, retrieval, quality, and value management, as well as reuse of research data [25]. Research data curation activities proposed in the literature include: discover, identify, select, obtain, verify, analyze, manage, archive, publish, and cite [28,29]. Each activity associated with data curation requires the use of different types of tools and metadata to describe, administer, and package research data [30].

Many models of research data and related curation activities and processes have been proposed in the literature. The Digital Curation Centre (DCC) from the UK proposed DCC Curation Lifecycle Model [31] and the Australian National Data Services (ANDS) designed ANDS Data Sharing Verbs [32]. They designed their models for the discovery and reuse of data and to provide flexible services in a heterogeneous data environment. In addition, there are many different community-driven tools for improving research data services and infrastructure. The Reference Model for an Open Archival Information System (OAIS) identifies an organization’s data assets and data curation tasks [33]. The Digital Repository Audit Method Based On Risk Assessment (DRAMBORA; [34]), the Data Audit Framework [35], and the Trustworthy Repositories Audit and Certification (TRAC; [36]) determine data curation activities, architecture components, and risks, as well as suggest policies for data archives and repositories.

As the number of curation-related models grows, many researchers and libraries have recognized the need for best practices in digital data curation [37]. Borgman, Wallis, and Enyedy [38] studied the data practices of a habitat ecology community. Stvilia et al. [29] studied the data practices of a condensed matter physics community. Wu, Worrall, and Stvilia [39] also explored the data practices of an earthquake engineering community. The study participants from those studies were scientists from the research communities. Large research university libraries also conducted case studies to understand the data practices of their institutional researchers [40,41]. In addition to studying data practices, the curation-related roles, skillsets, and trainings are also important research areas in the community. Kim, Addom, and Stanton [42] studied curation-work, -worker, and -workplace, as well as eScience professionals’ skillsets in order to develop a Library and Information Science (LIS) course curriculum. Also, a study found that many libraries are hiring new data professionals and/or shifting current staff into new data curation-related positions [8]. To the best of our knowledge, most researchers and libraries have explored data and data curation practices from the perspectives of data producers (e.g., researchers). Therefore, there is a dearth of knowledge regarding the current practices of IR staff in data curation-related activities, IRs’ activity structures, and role-related skillsets.

## Research design

Studying the practices of research data curation requires multifaceted contextual analysis [29,38,43]. Hence, this study requires a research design that can examine the sociotechnical and cultural factors that may affect data curation. The study was guided by an analysis of the literature on data curation and Activity Theory [44,45]. In particular, Activity Theory was used to conceptualize the general context of data curation work in IRs. This context comprises a system of different work activities and their structures, including different roles (e.g., providers, users, curators), types of data, tools and skills needed, rules and policies used, and mediation relationships among those structures.

To collect data, the study used semi-structured interviews. The target population of this research was the staff working for the IRs which stored and curated research data at the time of data collection. In this study, IR staff is defined as any person who participates in managing research data, from the point of its ingestion in an IR to its disposal, including maintaining it in an active state and enabling its discovery and re-use [31,46]. Participants sampled for the interviews had to meet the following criteria. First, they had to be involved in curation at their IRs. In order to qualify, their job titles did not necessarily need to be “data curator” or “repository manager.” Different institutions use different job titles, such as metadata librarian, digital repository manager, digital repository architect, digital curator, digital service librarian, scholarly communication librarian, research data librarian, data management librarian, etc. Second, participants had to work for IRs that stored and curated research data. Third, subjects had to work for IRs being maintained by one of the 108 institutions classified as RU/VH (very high research activity) in the Carnegie Classification of Institutions of Higher Education, a leading framework for recognizing and describing institutional diversity in United States higher education. The institutions classified as RU/VH provide more or less comparable sociotechnical contexts for IR operation, which may not be too skewed by possible resource disparities or differences in organizational missions and activities.

Purposive and snowball sampling techniques were used due to the difficulty of reaching or identifying the members of the population [47]. The study used key informants (e.g., heads of scholarly communication departments, IR software trainers, etc.) who had many connections in IR curator communities as sources to identify other members of the recruited sample. The researchers sent a total of 33 email invitations to potential participants. The email explained this study, the process of participation, the benefits and risks of this study, the participant’s rights, and included the study’s consent form as an attachment. Florida State University Institutional Review Board reviewed and approved this study, and the Assurance Number is IRB00000446. To minimize any possible risks or harms and to protect subjects’ welfare, researchers informed the purposes and the whole process of the research at stage of recruitment, so that subjects participated voluntarily. The main risk associated with participation was a possible inadvertent disclosure of private identifiable information that may damage participant reputation. The study employed thorough procedures to minimize this risk and protect participant confidentiality and anonymity at the extent allowed by law. Participants’ names are not associated with the content of the interview data. Participants had the right to have the recorder turned off at any time during the interview. Likewise, participants had the right to request the interview be stopped and notes destroyed. A total of 20 people responded the email invitations, but only 13 respondents met the sampling criteria. The 13 respondents agreed to participate in this study and sent an electronically signed consent form back to the researchers. Thirteen interviews with 15 participants were conducted. The participants were from 13 different institutions. Two of the participants each requested that an additional person be interviewed with them, which brought the total number of participants to 15. The interviews were recorded and transcribed. Each interview took between 55–80 minutes. The transcribed interview data were imported into QSR NVivo for Mac, a data analysis computer software, to conduct data analysis using the initial coding scheme developed by the researchers. One of the researchers performed an initial coding of the whole dataset. A second coder, who has experience in qualitative research and is familiar with Activity Theory, applied the same coding scheme to 10% of the transcribed interview data (i.e., 1.3 interviews) for quality control. The two coders had some disagreements in how they applied the coding scheme, but they discussed the differences and were able to achieve consensus. After the discussion, the first coder re-coded the whole dataset along the agreed upon application of the coding scheme.

## Findings

### Data curation activities

The interview data identified a variety of research data activities in IRs, including different curation activities as well as other related activities. Data curation activities mainly support the research data lifecycle (e.g., conceptualizing, planning, creating, uploading, and publishing); other related activities facilitate or motivate data management and reuse through IRs (e.g., data analysis, policy development, and education). Table 1 shows each activity type and its corresponding actions within the two major categories of research data activities.

**Table 1:** Research data activities and their corresponding actions in IRs.

**Curation Activities and Actions**	
**Activities**	**Actions**
Understanding data curation needs	Interviewing researchers
	Communicating with IR or library staff
	Consulting with researchers
Managing and sharing data	Receiving or transferring data files
	Cleaning data
	Converting data to a different file format
	Developing and adding metadata
	Validating data
	Packaging data
	Uploading and publishing data into IR
Ensuring that data is accessible and reusable	Annotating data for relevant entities
	Optimizing data to search engine
	Keeping data up to date into mirror repository
Re-evaluating data for long term preservation	Selecting dataset for long term preservation
**Other Related Activities and Actions**	
**Activities**	**Actions**
Analyzing data usage	Managing descriptive statistics of data usage
	Providing researchers with data tracking results
Creating policy and administrative infrastructure	Understanding local needs and creating local policies and rules
	Building infrastructure component
Educating people about data management	Training librarians
	Educating researchers
	Providing workshops for data analysis tools
	Providing outreach for data curation
Continuing education	Learning the best practices for research data management
	Learning future technologies

#### Understanding data curation needs

Most interviewees described spending a significant amount of time determining the extent of a data provider’s data curation needs. One of the interviewees explicitly emphasized the importance of the first meeting with a data provider: “I think how much assistance each researcher needs really depends on the first interview. You are getting to know how well they have organized their data.” (subject4—s4). First meetings with data providers to assess their data and curation needs notably affects later activities, such as receiving data and creating metadata. The meetings determine what types of help the data providers need, identify the right person to help them create metadata, and discuss how the data could be organized and stored:

“I specifically meet with researchers who are interested in actually depositing their datasets into our repository. So, I am the first staff to talk about what we have and get to know their data needs, and then bring in other specialists in our library, such as metadata librarians, who would be involved in the project. We get them into the conversation once it is appropriate.”

(s4).

In addition to communicating with researchers, dialogue with other IR or library staff also helps IR staff connect the right person to each researcher in order to address the issues detected in the first meeting; this involves “Going and meeting with people, getting people to coordinate work, and talking to people about understanding each project” (s1). Interviewees also consult with researchers to provide support for the researchers’ data actions (e.g., depositing, documenting, and organizing). When IR staff are asked for help with those actions, they actively work with researchers to enable better access to datasets stored in IRs:

“We are actively working with researchers to do the deposit, and this is happening more around research data. We are actually doing work consultation on how to describe their research data, as well as how to organize it for download purposes, and, also, for segmenting it into particular files, especially for larger datasets. I guess essentially to enable better access to those datasets.”

(s5).

Interestingly, one interviewee indicated that his IR has a very strong emphasis on communicating with researchers about their projects. The IR staff endeavor to keep in touch with the researchers periodically in order to stay current on each project’s status (e.g., grant proposal, reward, creating data, depositing data, publishing data):

“If we were to go from the beginning we would be the first people to meet with researchers when they were interested in archiving data. It might be part of when they’re working on a proposal or at a later date. We try to ideally meet with them in person and discuss what they’d want to go in and inform them of our protocols and such. We keep in touch with them. If it’s a grant proposal, we keep in touch of whether or not they get their reward, and when they get their reward we try to contact them right at the beginning of a project…. Then periodically we would keep in touch with them. In other cases we’ll get someone at the end of a project where they just have data for us so we can meet with them and go over the process and give them some tips on how to organize their data. When they are ready to transfer data, in most cases we like to meet with them directly and have them transfer data right to a portable hard drive.”

(s14).

#### Managing and sharing data

This activity typically begins with receiving or transferring data files. Data providers generally deliver their files through file sharing services or portable hard drives:

“I do file transfer, so in some cases, if you have a large file set, too many files, or it's too big size-wise, our work is to transfer either through a file sharing service or by going to somebody's office with a hard drive.”

(s15).

Once the IR staff receive data from researchers, they either help researchers clean up their data or the staff clean it themselves. One of the interviewees mentioned that they specifically work on discrepancies in how the researchers’ names are spelled: “We do some metadata clean up. People tend to submit things with their name spelled different ways. We do some metadata clean up for that.” (s6). An interviewee stated that they also clean data from a disciplinary perspective (e.g., cleaning variable headings, organizing file directories): “I would probably help earlier on with getting their data cleaned up from the sort of discipline side of things [like cleaning] basic variable headings, file organizations, all that.” (s4). Converting proprietary file format into non-proprietary format is a prevalent action in the interviewees’ IRs. Some of the data files need their formats converted because they use fairly expensive software. Since it is important to be able to reuse, share, and preserve data, employing inaccessible software is not an ideal choice:

“We do recommend that they try to convert proprietary formats into nonproprietary when they can. We have some cases of that. There are a few where they used plotting software. It was called sigma plot. To actually use the file, someone would need fairly expensive software to get at it. In that case, we took the time to export things to just standard tables… It ended up taking a lot of time because it didn’t transfer the role headings and metadata; the process of copying all that took more time than what we can really do in a lot of cases.”

(s14).

A different but related issue is the need to convert files into flattened file formats. Some research data files have more than one type of data. For example, a spreadsheet may also contain screenshots of image data generated by proprietary software. In such cases, creating the metadata and characterizing the file formats are not simple tasks.

“One of the things I have seen that really gets complicated is what people do to record their research. They do what they have to do. Sort of make their research more efficient. I met with someone who was doing medical research, and he had an Excel file. He did complicated micro-species research. [The file contained] thousands of images he was creating, and he would then ingest those from proprietary analysis software to create figures that he could use to analyze data, and he also could use them for publication. So, he actually took a screenshot of the proprietary software and then embedded a JPEG of that screenshot into an Excel file. He created extremely complicated digital objects that if you are trying to flatten them it would be very hard.”

(s1).

IR staff also help researchers develop metadata for their research data. They may add not only descriptive metadata, but also administrative and preservation metadata:

“I do the initial ingest process. What we do is a virus check; we do integrated scanning format checking. I help develop metadata for the data that's both descriptive metadata and also administrative and preservation metadata in terms of what we've done with the data. I put the data into a consistent package and upload it to the repository and again do things like integrity checking in virus scanning before the data goes into the repository.”

(s15).

Another interviewee also mentioned that he helps researchers document their data. Even though researchers have their own understanding of metadata, they need further assistance to fully describe the data in the ways that they want it described:

“In my position, a lot of the work I do with researchers contribute to that metadata piece. Because even though they are describing, they are also creating a record for digital objects very similar to what you see in a published online journal article. But a lot of the time, they want someone working through the process and [then we] help them with the stages of the data [until] they get there. To describe their data in the ways that they want it described.”

(s3).

Researchers’ different interpretations of metadata schemas requires intensive conversation between IR staff and researchers in order to create appropriate metadata:

“Actually, we do a lot of back and forth with them. They want to know that things are going to be exactly what they want them to be. Kind of learning the system; learning what they can or cannot do with presentation of metadata or metadata record. That's why I have a lot of interactions and conversations, sometimes in month-long conversations at the stages of dataset. It is more than the human service side of things, in that respect, because the metadata form, DC [Dublin Core] term, is very straightforward. However, when people present with the form, they interpret it in many ways. Even though it is straightforward.”

(s3).

Most interviewees talked about helping researchers create a Read Me file within a dataset. Because of the limited set of descriptive metadata elements IRs provide, researchers often want to add supplementary information about the data by using a Read Me file, a sort of “workaround” [48]. The file typically contains more specific information or disciplinary information about the data, along with different metadata schemas or vocabularies. The files tend to be only relevant to the domain specialists of that data due to the specificity of the information:

“Whenever we acquire a new dataset, typically from a faculty member, we work with them to describe the dataset using a qualified Dublin Core set of metadata. That gives us a generic bibliographic record for the content. We then work with them to create a Read Me file for the dataset which gives more specific information about the dataset that may use different vocabulary that's more focused on the discipline and include other kinds of information that are really applicable only to the users of that data.”

(s10).

In addition, a few interviewees directly stated that they write metadata for the data researchers provide. While creating metadata, IR staff spend a significant portion of their time collecting documents that may help them understand the research data:

“When the researcher has time, we do try to sit down and interview them to talk about what they did, and we write the narratives, not a full transcript, but we do produce a narrative of that conversation. If they had NSF [National Science Foundation] funding, I try to get the proposal that they have in IRB [Institutional Review Board]. I get the IRB and we will pass them or look up for ourselves a kind of representative sample of publications based on their data, so that we know something about it; we know the context of it, and so, based on all those things, we will write a lot of metadata ourselves. And so basically, I would say 70–80% of the time, it's just accumulating documentation.”

(s15).

Before a research dataset is uploaded into a repository, there are two more steps to do. The first is data validation, which checks whether the data contains any errors in its content. An interviewee provided an example of this:

“We talk with them and make sure the data is represented in the way they want it to be. We help take a look at [their dataset] like a tabular dataset and ask questions like, ‘Are there supposed to be no values within those or should they all be zeroed out?’ and they may have a very good reason for what they are doing.”

(s10).

The second step is preparing the data package. Some datasets contain multiple files, and the files are different types (e.g., image, text, audio-visual). Most IRs require the depositors to submit a zip file package instead of submitting each file individually:

“Usually we just package the dataset with supplementary information like a data dictionary. Like in a zip package with the dataset and any other documentation.”

(s1).

The last action for depositing data is uploading and publishing the data into an IR. Some of the interviewees mentioned that their curation team had the researchers give data to them, and then they would ingest data into their system. In addition, an interview participant described a specific service his institution supports that can be used just before publishing the uploaded data. The service allows researchers to look at their metadata in a test view and enables them to have one last opportunity to edit the metadata and its supplementary information:

“We upload everything onto a test instance that’s not online in our IR platform so that we can share a link with just them [researchers] that’s not public yet. They get to look at the metadata and can make final edits. Then, if they approve it, we move it to our regular online instance.”

(s14).

#### Ensuring that data is accessible and reusable

IR staff aim to ensure that data is accessible and reusable online after the data is deposited and published. Some of the interviewees introduced different efforts to improve the accessibility and reusability of the data (e.g., managing metadata for search engine optimization, managing and maintaining the links between a central repository and a mirror repository). Similarly, an interviewee presented an effort to create maps connecting researchers’ names with their affiliation information. The mapping may not only improve reusability of the data, but also reduce ambiguity regarding researchers’ names:

“We do some mapping from collection, because as you are aware not all researchers are housed in one department or research lab. So, someone is in one research lab, but they are also in another department. I want to map things from place to place, so they can be found.”

(s6).

#### Re-evaluating data for long-term preservation

A few of the interviewees shared that they conduct re-evaluation activities based on their preservation policy, but the re-evaluation has never actually been started and completed before. Their IRs have not had a long enough period of operation to do re-evaluation activities. Re-evaluating data is for long-term preservation; select IR staff, such as archival specialists, subject specialists, and IR managers evaluate the data to see whether they should be preserved or deselected. In order to be re-evaluated, the data must have been stored for the duration specified in the IR’s data retention policy. Five or ten years were the examples described in the interview data. An interviewee presented his institution’s policy: “We have a collection policy [to] dictate what happens to [data] 10 years after the deposit.” (s3).

#### Analyzing data usage

Many interviewees described analyzing data usage as one of their data activities. They manage data statistics for different purposes: (1) to facilitate their own administrative work: “We manage statistics to try to understand the number of downloads.” (s5). And, (2) to provide tracking results to the content providers: “Over time we will be giving the researchers tracking results on how often their data has been downloaded.” (s14).

#### Creating policy and designing and administering system infrastructure

Policy development and system improvement take time to be built up to a satisfactory level that follows current state of the art practices and harmonizes with other local policies. IR staff specifically needs to understand the recommended policies, rules, and norms around data management, and be able to select the policies that they want to adopt. In addition, the policies from external sources must align with current local policies or rules to produce successful results. One of the interviewees talked about the process of policy construction:

“We are trying to figure out data retention policies, how to interact with collection development policies, and institutional policies for research data management that are being communicated now. Even though we are accepting stuff [research data] into the system, it takes a long time to create the policies. So, we are working in parallel.”

(s1).

Developing data standards and policies is a political process, which may involve negotiation among different stakeholders, evangelism, and persuasion of colleagues and opponents [49,50]. As one participant revealed, sometimes IR curators have to start with norms advocated and enforced by an individual staff member knowledgeable about a specific data management component rather than with a formal policy.

“To some extent, there are policies or rules, but identifiers and identifiers on the web has been an area of interest of mine for a good 15 years, so to some extent, I was vocal about what I thought we should do and I think a lot about what we should do…. so that maybe the only rules or norms were the ones in my head that I kept repeating at people.”

(s12).

A similar experience an interviewee described is the process of designing and administering system infrastructure. Building and managing the system is a highly time-consuming activity for IR staff: “I've been responsible for a lot of the infrastructure components and a lot of the thinking about how the pieces of this come together.” (s10).

#### Educating people about data management

Managing research data is a new area of study in information organization. The community practices are still developing and academic libraries are accumulating this growing knowledge. However, there are currently a variety of teaching activities associated with the IRs in this study. First of all, IR staff teach the librarians about IR resources so they can communicate that information to library patrons, including both students and faculty members:

“We are training all the librarians and people that are in reference desks and everyone in the library who deals with any of our patrons, any of our faculty. To make sure that everyone does really know, we have an IR. Your data can be open accessed, and if you have bigger data or sensitive data, whether it's HIPPA sensitive or privacy sensitive, here is our website that can explain it to you, and here are the different contexts you can have. So you can always come through us.”

(s11).

Second, IR staff design events to provide research data management practice for their campus communities. The events aim to educate researchers in data management, as well as answer any of their data-related questions:

“I’ve done some campus-wide events. We’ve had ones on big data, little data, and having all of that. We do a research-computing day once a semester. We’re also looking at doing a ‘bring out your data’ event where we have a bunch of data experts… A whole bunch of people in the same room where people can just come and ask everyone at once about their data needs. I set up a lot of events like that. We need to make sure the right people are in the room”

(s11).

The third type of data management education focuses on how to use IR platforms and data analysis tools:

“I suppose a lot of work that we do is educating users how to use the [IR] platform itself. We, like many IRs, don’t have a lot of self-in [systems] to do that kind of content ingestion on behalf of users. So, there is a lot of trying to help people manage data by themselves.”

(s2).

One of the interviewees also mentioned teaching users how to implement data analysis tools: “We do provide workshops on data analysis tools for users.” (s15). The last education type focuses on IR staff providing outreach to promote the use of research data curation services within their IRs:

“We have done a multi-media campaign starting last January. We did a postcard mail out too, we distributed postcards across campus, provided multiple workshops internally for librarians, and multiple workshops externally for data management planning IR use.”

(s3).

#### Continuing education

IR staff learns the best practices, policies, rules, norms, and technologies of research data management services from data curation and archival communities, which they can then use to develop their own IR systems. Because data curation is an emerging area of practice, IR staff accumulates knowledge from different communities, in order to ensure both the effective processing and long-term preservation of research data. More specifically, they learn by attending conferences or data curation-specialized trainings, taking coursework, reading articles, and benchmarking the practices of peer institutions:

“The best practices come from the data curation community and the archival community, and policies and rules are being created now locally for our system.”

(s1).

“Some of the best practices are sort of what we learned in conferences or in training or coursework we are taking. We look at universities that have been at the research data management game for a little bit longer than we have. So…. looking to see what they are suggesting for best practices. Kind of combined those things into our local setting.”

(s1).

In addition, IR staff collaborate with research and technology experts on their campuses to prepare for future changes in IR systems: “Liaising with researchers, computing people, and our research computing advisory committee to look at how we build up the technologies that are needed for the future.” (s11).

### User activities

Users’ roles around research data activities in IRs could broadly be divided into two groups: (1) data providers and (2) data users. Each group is a group of people who share the same objective of uploading and/or downloading research data trough IRs [44].

#### Data providers

Data providers are mostly researchers: faculty members, postdoc researchers, and graduate students in each institution. A few institutions also allowed undergraduate students to submit their data with faculty advisors’ permissions. A main activity of data providers in relation to IRs is the submission of data files and their metadata (Table 2). There are two different types of submission processes. First, researchers provide their data and its metadata directly to IR staff: “The users provide us with the data files and spreadsheet that contains all the metadata that they want to import to the repository.” (s2). Second, researchers upload their data and the metadata into the IR systems: “You upload your files for publication phase. And then using a wizard… you can contribute metadata so you can describe the dataset.” (s3). The second main activity identified is the conversion of file formats. In most cases, data providers consult with IR staff, and the staff recommend nonproprietary file formats for long-term accessibility and preservation: “We do recommend that they try to convert proprietary formats into nonproprietary when they can.” (s14). In addition to those two main activities, some of the interviewees described activities associated with services provided by IR systems. Using IR services, data providers are able to track their research data’s usage and number of downloads:

“The researcher can create their own profile they call selective works I guess. So, there is a sort of dashboard you get, and when you sign up for that it allows you to look at downloads and track what is happening with the publication.”

(s1).

**Table 2:** Data providers and their activities.

**Data Providers**	
Faculty members, postdoc researchers, graduate students, and undergraduate students	
**Activities**	
Provide data files and its metadata	Share data through social networking sites
Convert file formats to nonproprietary formats	Share recommended citation and contribute citation data
Track data usage (downloads, publication uses, etc.)	Collaborate with researchers in IR project space
	Transfer data ownership from student to faculty advisor

Sharing activity through social networking sites is also supported by IR systems: “We have, through the IR, a social networking widget that allows you to tweak [share] the paper or data.” (s1). Data providers can share a recommended citation and build citation information for their data:

“For every item in the repository, we have a suggested citation if you want to quickly share a citation. You can just copy and paste a generic format citation if you don’t have a very specific format you want.”

(s10).

Another interviewee mentioned IR services associated with citations: “We provide recommended citations and allow the contribution of citations. Or you can link to other digital objects or just provide citations to non-digital resources.” (s3).

There are some activities that were only provided by a single IR. The first one is providing IR support for collaboration within a project team. Project team collaborations are facilitated by using wiki capabilities: “You can upload your files [in the project space]. It has a wiki capability. You can assign tasks, which is a project management functionality” (s3). The second activity available for data providers at one IR is the ability to transfer ownership of their datasets. This activity is important because it enables graduate students to transfer data ownership to their advisors:

“Users can also allow another user to deposit on their behalf… That user can also transfer ownership back to the owner of the file. There is an ability to let somebody else, like a graduate assistant for a faculty member, let’s say, deposit files on his or her behalf and then transfer the ownership of those files once that has been done.”

(s13).

#### Data users

Data users of the IRs can be anyone who has Internet access and is interested in the stored research data. The activities of the users are dependent on the IR systems (Table 3). There are two different types of IRs: (1) IRs that only provide typical repository services (e.g., identifying, searching, browsing, and downloading): “We support searching, browsing, download of materials.” (s5); “We don’t actually offer any direct tools that do [analysis]. We make our data available to the end users and we pretty much expect them to take that data and move it into their tool of choice.” (s10); and (2) IRs that provide not only repository services but also diverse user services. Many interviewees mentioned the sharing and social networking ability of their IRs:

“Our dataset is searchable. You can browse as you see. There is a social networking ability, where you can disseminate a dataset, in the upper right corner of every dataset record. You can select how you want to distribute the dataset using Facebook or Twitter, for example.”

(s3).

**Table 3:** Users’ data activities in IRs.

Users’ Activities through IR User Services	
Typical Repository Services	Additional Services by the IRs
Identifying	Sharing
Searching	Social Networking
Browsing	Full-text Searching
Downloading	Bookmarking

Some other interviewees also talked about full-text searching and bookmarking services:

“We have mechanisms to search for data and to browse through items in our repository. They can search the full text of the items. They can share items that they found with various social networking platforms.”

(s10).

“We also have the bookshelves…. I think people normally save their favorites and save their items elsewhere, whatever their other external system is, but people have really used those [bookshelves] a lot, especially in teaching.”

(s11).

Some of the interviewees from the IRs with only typical repository services explained why they decided to not provide additional services. They assumed that the users would already have the tools to work with the data:

“Honestly, we don't really provide much of anything. I mean our data is available for download. So the assumption is that users would have the tools necessary to interact with it. I am actually situated with a unit that does a lot of work around numeric data analysis, GIS [Geographic Information System] data, as well as qualitative data analysis. But you know that's a separate unit, not explicitly connected with the IR.”

(s5).

Some of the interviewees also thought that an IR is simply meant to be a repository, not a user interactive site:

“Repository services. I actually advise users to use alternate systems, whether it’s Google or something else, just to get access to the file. It’s a repository, not a user interactive site.”

(s9).

### The activity structure

The structure or context of research data activities in IRs consist of objectives, goals, division of labor, data types, tools, policies, rules, and norms [44,45]. This section describes the activity structure identified by the interview data.

#### Objectives

The main objective of the IRs is to collect, access, store, preserve, and share research scholarship, as well as other materials that reflect the intellectual life of the university. All of the IR staff who were interviewed mentioned similar obejctives. An interviewee explained that collecting research output in the university is one of the main objectives of his IR:

“Like a lot of institutional repositories, part of what we want to do is to capture the scholarship and research outputs in the university, whether that is publications or datasets, so that one of the primary objectives is to have pre-prints or post-prints, and wherever possible the actual published version of the faculty member's work, within the institutional repository.”

(s15).

The preservation and accessibility of scholarly output through IRs were also presented by one of the interviewees as goals: “I see the main objective of our repository service as making accessible and preserving the scholarly output of our research communities.” (s13). Lastly, an interviewee described IRs as providing data storage and long-term preservation, while also enhancing the circulation of data among researchers:

“It is housing research datasets, but it is also part of the digital collection managed by the library. So, this IR is incorporated with the existing library collections, and so, as far as the research dataset side, the main objective is basically to provide both data storage and preservation to those who produce research datasets on campus, and also to increase data sharing by researchers.”

(s4).

As an optional function, a few interviewees introduced the idea that one of their main objectives is to publish open access journals through their IRs:

“The main objective of the IR is twofold. The first is to host and make available publications from scholars at the institution, and the second, which is a growing element of the IR, is to actually publish open access materials, primarily journals.”

(s1).

In addition to the general objectives of the IRs, some interesting objectives regarding research data curation were identified in relatively new IRs. These newer IRs were built after January 2011 when NSF required its grant applicants to submit a research data management plan [4]. The institutions had mainly developed new repositories in response to major funding agencies’ policy changes on research data management. Five out of 13 IRs were developed after January 2011. The IRs’ services started with a research data focus:

“We developed [the IR] in 2012 after never having had a repository service, which made us a little bit of an outlier for a big research university. You might be wondering why we did it in 2012, and a big part of the answer was, it was in response to the NSF data management plan requirement.”

(s12).

Two of the five IRs created after 2011 were designed to support the entire research data lifecycle (e.g., DCC Curation Lifecycle Model, OAIS), from planning and creating to publishing and disseminating their research data on the Web:

“We want to support the entire research data lifecycle from data management planning, grant application, initial research project, data staging, virtual research environment for collaboration through publication with DOI, dissemination of data openly on the Web, and finally, this is a really important piece, preservation.”

(s3).

Additionally, the departments supporting those IRs provide consulting services, instruction in writing grant proposals, and data management training for their content providers in order to support a holistic approach to research data curation:

“Our department grew services for archiving through the [IR] software, and then also has additional services around consulting and preparing data management plans for grant proposals or other data management training in general.”

(s14).

#### Division of labor

IR staff are the employees who work for IRs, and their job responsibilities involve curating or managing objects within an IR. IR staff, based on the interview data, can be divided into seven different roles: (1) head, (2) data curator, (3) IR manager, (4) metadata specialist, (5) developer, (6) subject specialist, and (7) graduate assistant. Table 4 is a map of the relationships between the roles and the position titles that exist in the interviewees’ IR staff. The positions of data curator, IR manager, and developer exist in almost all IRs. On the other hand, the positions corresponding to head, metadata specialist, subject specialist, and graduate assistant only appeared in less than half of the 13 IRs. Some of the IRs only had one person to perform multiple roles of IR staff. For example, three different cases were identified: (1) head, data curator, and IR manager, (2) head and data curator, and (3) data curator and IR manager.

**Table 4:** IR position titles mapped into identified IR staff’s roles.

Roles	Job titles that include a particular role	# of IRs that have the roles
**Head**	Head of IR, Director of Scholarly Communication, Head of Digital Publishing, Assistant Dean for Digital Libraries, Head of Publishing and Curation Services	6
**Data Curator**	Data Service Librarian, Science Data Management Librarian, Repository Specialist, Technical Analyst, Repository Coordinator, Data Curation Specialist, IR Coordinator, Data Librarian, Curation Librarian, Digital Scholarship Librarian, Digital Collections Curator, Digital Content Strategist, Data Management Consultant, Data Curation Librarian, Digital Projects Designer	13
**IR Manager**	IR Manager, Repository Specialist, Repository Coordinator, IR Coordinator, IR Production Manager, Data Management Consultant, System Administrator, Digital Collections Curator	12
**Metadata Specialist**	Metadata Specialist, Head of Digital Project Unit, Digital Metadata Head	3
**Developer**	Developer, IR Administrator, System Administrator, Technology Architect, Software Developer, Senior Computer Specialist	13
**Subject Specialist**	Collection Administrator, Subject Librarian, Community Administrator, Subject Specialist	5
**Graduate Assistant**	Graduate Assistant	3

The role-related activities of head positions included three different tasks. First, they plan and build their research data IR services and further design the services to fit within their library systems. As the lead person of an IR group, they are responsible for planning and building their infrastructure:

“[This] is an evolving position. I think what we’re trying to achieve by having that position is to develop a holistic view on the way that we are developing our digital collections, including what goes into our IR, but also thinking holistically about collections, so not making the digital collections like a silo, away from the other kinds of collections that exist in the university or that the libraries collect.”

(s13).

In addition, the heads of IR groups communicate with researchers to address their concerns and answer their questions regarding IR use. They also provide outreach services to encourage use of their IRs (Table 5):

“The other piece of that is providing user services, so being the person that works directly with researchers and hearing from them about what their requests are, what the challenges are in using our IR, and doing a lot of outreach for the IR and that kind of thing.”

(s13).

**Table 5:** IR staff’s role-related activities.

Roles	Role-Related Activities
**Head**	Build or plan data governance structure in their IR
	Communicate with researchers
	Provide outreach for their IRs
**Data Curator**	Consult with data providers and connect them to metadata specialists or IR managers
	Facilitate communication across different entities
	Evaluate or view research data to see whether the dataset would continue to be maintained or whether it would be deselected
	Build or plan data governance structure in their IRs
	Outreach and educate campus community
**IR Manager**	Manage IRs on a daily basis
	Work with data providers to help add metadata and upload data into IRs
	Answer questions about IR use and data management
	Outreach and educate campus community
**Metadata Specialist**	Help data providers to create appropriate metadata for their dataset
	Design metadata schema for their IRs
**Developer**	Maintain and update IR software
**Subject Specialist**	Evaluate or view research data to see whether the dataset should continue to be maintained or whether it should be deselected
	Manage and approve incoming submissions to their own collections
	Provide support and help to the management of the IR from their subject/user community specific perspectives
**Graduate Assistant**	Assist data curators or IR managers

Unlike the position of the heads, which was found at only six of the institutions, all of the interviewees mentioned that they have one or more data curator in their IRs. As the name implies, the responsibilities of data curators include numerous activities, some of which partially overlap with the responsibilities of the heads. One of their primary responsibilities is consulting with data providers to understand and address their needs, which is often accomplished by connecting them to the right person:

“We have a subject specialist-centered service model. So, each time I am in contact with a researcher, each time they create a project in our IR, each time they submit a publication, or when they have a successful grant application or where they used our IR in their data management plan, I would like their subject specialist librarian to know, and, the three of us, in a perfect world, work together to get them to use our IR.”

(s3).

Another interviewee also described a similar work model in her institution. That work model motivated the interviewee to teach librarians about research data management:

“I can direct them to others or they contact their librarians, any of the subject specialist librarians. So, we have to make sure that all of them are ready for answering those basic questions on the IR about research data support on campus.”

(s11).

Data curators also play the role of facilitator between different work units. Specifically, the main area of responsibility is facilitating technical communication among stakeholders:

“I think of myself mainly as a translator because I have the depth and breadth of the technical knowledge, as well as the academic research practices, knowledge and means, as well as the library practices, needs, and technologies. There are a couple of people on campus like me.… Sometimes other people call us “glue people.” We help things connect and stick together.”

(s11).

The curators also collaborate with librarians to select datasets for long-term preservation. Not all of the datasets are appropriate for permanent storage. They select datasets for preservation through the lens of archivist, subject specialist, and data curator:

“Datasets would be viewed by digital archivists, subject specialist librarians, and digital data repository specialists to see whether the datasets should continue to be maintained or whether they should be deselected.”

(s3).

More than half of the institutions enabled data curators to perform typical responsibilities of the head position. Like the heads, the curators plan data governance structure for their IRs, and follow the current and future trends of the research data curation community:

“I do the next thing, which is considering how we leverage the repository information structure in order to support changes in library publishing, changes in data curation, changes in new forms of digital scholarship, alternative scholarly work, and so on.”

(s11).

Outreach for the IR services was also a widespread activity throughout the IR staff, including head, data curator, and IR manager.

A primary role of IR managers is managing IRs. Simply put, they manage IRs on a daily basis: “She [IR manager] does a majority of day to day managing [of the IR].” (s2). They ensure the ongoing production flow of adding new materials to the IRs. Along with managing IRs, they answer any questions regarding IR use:

“My primary job is to be the frontline person for our IR. I handle any incoming questions about the IR, requests for consultations, and demonstrations about the IR.”

(s3).

IR managers also communicate with data providers to create and add metadata, and to upload the data into IRs:

“Major role.… is helping users [data providers] provide and add their metadata for their research project and curating. But, I also communicate with them to provide interaction between what they want and what I want.”

(s3).

Another responsibility of IR managers is to provide outreach for their IRs and educate the campus community. These activities are a shared responsibility between the head, data curator, and IR manager roles: “[I do] any internal and external outreach for education, demos, and troubleshooting questions in terms of when someone actually runs into a bug or issue using the IR” (s3).

A few interviewees mentioned a separated metadata specialist position within their IR staff. Those interviewees indicated that there is a high demand for metadata specialists:

“Our metadata staff is currently influx. We have one digital metadata head, who is very much involved [in the data curation team]. She is a key player for sure. We also, in the process, put out the position and hiring process.…. We are going to have one full metadata specialist working on our research data curation team and also interact with the researchers to get their data documented for and deposited into our IR.”

(s4).

The complex and diverse types of research data also required the continuous design/redesign and maintenance of metadata schema for research data: “[The metadata specialist] helps a lot with the metadata schemas and modeling as we add new content into the repository.” (s10).

All of the 13 IRs included one or more than one person working as the IR software developer. They generally maintain their IRs and update the system as needed:

“We also have systems people or library system staff help manage the backend [systems], make sure all the backups are there, do upgrades to the software, etc.”

(s9).

In addition to that, one of the interviewees mentioned that they purchase a developer service from an outside company: “Technical support comes from an external company; we are purchasing services from them.” (s1).

Almost half of the interviewees referenced subject specialists connected to different departments. They can be subject librarians working closely with different departments or people who have been designated by the department to administer their own departmental collections. One of the responsibilities of subject specialists is evaluating research data for long-term preservation. As mentioned in the description of data curator roles, the evaluation is co-conducted by an archival specialist, subject specialist, and data curator. The primary tasks of the subject specialists draw on their domain knowledge. They manage departmental collections, approve incoming submissions to their own collections, and help manage the IR from their specific disciplinary perspectives:

“These are the people who have been designated by the department, or by the research group to manage and approve incoming submissions to their repositories, and also oftentimes make deposits themselves.”

(s2).

Another interviewee also spoke about the responsibilities of subject specialists:

“All of our subject specialist librarians connected to different departments inform and provide support and help to the management of the IR from their specific lens and their constitutive perspectives.”

(s11).

One of the interviewees assigned a wide-ranging set of tasks to the subject specialist role in her IR. In that IR, the subject specialists can establish disciplinary workflows, submission processes, and setup a new collection:

“We also have community members [in a department, institute, or research center, etc.] who are actually able to manage a collection within our IR. We give them appropriate permission so that they can establish the workflow and submission processes they want, or they can setup new collections, they can setup groups of submitters, and they have some level of control over the entire repository. So, we call those community administrators.”

(s5).

A few IRs had graduate assistants who assist data curators or IR managers. Their responsibilities are very flexible based on the tasks set by their supervisors: “I have a limited amount of graduate hourly support. There is a graduate student who works for me on an hourly basis to complete tasks.” (s5).

#### Data types and their entity types

In response to the interview question, “What major types of research data does your IR accept?” all of the interviewees stated that they accept any type of data. They tend to accept any file format that can be downloaded. One of the interviewees explained:

“We’re able to accept just about anything in the digital format that they want to include, as long as it can be downloaded. Something like a database file would be downloaded just as a database file; we don’t have any interface for them to use it like a database online. They just download the file and run it on their own software.”

(s14).

Another interviewee also mentioned that they accept all types of data: “We accept all types. We’re pretty non-admonitory. We currently have a lot of raw data, text documents, spreadsheets, and SPSS [Statistical Package for the Social Sciences] files.” (s9). One interviewee even said that if data providers want to share it, they will put it in the IR. Table 6 shows the major types of research data deposited in the IRs, as well as some criteria for data-type limitation. The responses to the question on the major types of research data accepted into the IR led the researcher to make one additional interview question: “Is there any limitation regarding the file types that you accept in your IR?” Many interviewees answered this question with interesting responses. Some of the interviewees mentioned that the size and the number of files are restricted. One of them stated:

“We can't accept a terabyte of research data all at once. Also, because the only sort of interaction with the items is through downloading and uploading, you are restricted by the size of the items in that way as well. So, you can't add items that are just too big to download or too big to upload. So, size is a big restriction.”

(s5).

**Table 6:** Major types of research data and their entity types.

**Major Types of Research Data**		**Criteria for Data File Properties**
Any types of data (e.g., Raw data), Text documents (e.g., Word, PDF, LaTeX, TXT), Spreadsheets (e.g., Excel), Slides (e.g., PowerPoint), Audios, Audio-Visuals, Images, Laboratory Notes, Statistical data files, Databases (e.g., Access, MySQL, Oracle), Software codes, Tabular data files		File Capacity, The Number of Files, Proprietary Files Extension (e.g.,.exe)
**Entity Types**	**Metadata Elements**	**Identification Schemes**
Intellectual Entity	Title, Main Title, Other Title, Abbreviated Title, Subtitle, Abstract, Grant, Citation, Supplementary Information, Description, Material Type, Language, Target Audience, Reviews, Open Summary, Subject Summary, Identifier, Related URL, Right	DOI, ARK, Handle, HTTP URI, Permanent Local URL
Object	Title, Identifier, Related URL, File Format, Description, Supplementary Information, Note, Citation	DOI, ARK, Handle, HTTP URI, Permanent Local URL
Symbolic Object	Title, Identifier, Related URL, File Format, Description, Supplementary Information, Note, Citation	DOI, ARK, Handle, HTTP URI, Permanent Local URL
Person	Author, Creator, Contributor	Local Name Authority Records, ORCID
Organization	Larger body of work, Publisher, Source institution, Physical Container, Funder	Local Authority Control System
Place	Place of Publication, Holding Location, Spatial Coverage, Coordinates, Physical Container	GeoName Database (GeoNameID)
Time	Date, Publication Date, Copyright Year, Temporal Coverage, Time	
Event	Process, Publication Status, Edition	
Topic	Subject Keyword, Methodology, Genre	LCSH, MESH, and FAST with HTTP URI

Another interviewee added that the number of files is also a criterion of file-type restriction:

“We have a mirror [repository] for some of our research dataset. If it's too big for our repository software and if there are too many files for our IR platform, we create a shelf record in the platform and then we have a link out to the files in the mirror. One of our datasets is almost a terabyte, something like 5000 files.”

(s15).

Proprietary file extensions such as.exe could be a restriction, but only one IR actually restricts the file type. Most of the other IRs allow researchers to submit the files, although they recognize the problem that comes along with the file type and recommend not using the file format. The interviewee of the IR that restricts proprietary file types explained: “We don’t allow people to upload.exe files directly. They have to zip them because we don’t want anyone to have an.exe file on the server. Someone else could download it and have a problem on their machine.” (s11).

The interview data identified some metadata elements that are offered by the IRs. The elements were analyzed and categorized by the research data entity types identified by the researchers’ previous study [43]. Table 6 shows the categorization and identification schemes used for the entity types. All of the metadata elements could be mapped into one or more entity types, but many of the interviewees indicated that they frequently discuss the relationships between these abstract entities: intellectual entity, object, and symbolic object. The discussions primarily focus on selecting an appropriate entity level for a dataset when IR staff organize and deposit the dataset into the IRs. The IR staff could deposit a dataset into a collection level (i.e., intellectual entity), and they also could deposit each object of the dataset into an object level (i.e., object entity or symbolic object entity) along with different metadata. Currently the staff make the decision according to their communication with the data providers. However, in many cases, data providers just rely on the IR staff to decide. One of the interviewees mentioned that they deal with those kinds of discussions on a daily basis:

“One of the things that has been somewhat tricky and we’ve always tried to help researchers with is trying to find that right level of description for how things are grouped. Sometimes, we will actually create at the intellectual entity level a separate collection in DSpace, just because it’s easier to group the different objects under that intellectual entity. Sometimes, the objects will have different metadata, but they’re still one intellectual unit for the purpose of the dataset as a whole.… Those three actually [intellectual entity, object entity, symbolic object entity], it’s interesting. They’re tricky concepts, but we deal with those on a daily basis. They’re very much a reality when you’re a researcher with a bunch of data in front of you, trying to figure out how you want to group the data.”

(s9).

Another interviewee also indicated that there is no systematic recommendation or guideline for this issue:

“Within the system, for us, any of those [datasets] can be modeled either as individual items, if you, as a researcher, wanted to deposit a number of research data samples as a single entity [intellectual entity], or as multiple items [object or symbolic object]; it could be done either way. It really just depends how much time you want to put into modeling a collection. … once again, depends on how you want to formulate it as a researcher, and we try to allow for multiple representations, if that’s how you [the researcher] want. We typically have a conversation with the researcher to understand what they’re trying to accomplish with it. Many times, they don’t care. They just want us to tell them how to do it; however, sometimes they have a preference for it and so then we try to accommodate that if possible.”

(s10).

All of the entity types except time and event entities are identified by at least one of the different identification schemas (Table 6). Among the schemes, identifier schemas (i.e., DOI, Handle, ARK, HTTP URI, Permanent Local URL, and GeoNameID) identified the entities of intellectual entity, object, symbolic object, place, and topic. The identifiers that can be assigned to datasets could identify the first three entities. Place entity can be identified by GeoNames database (i.e., GeoNameID). Topic entity is specified by different subject lists that are supported by the linked data technique (i.e., HTTP URI). One of the interviewees mentioned how they use identification schemes for different entity types:

“We have a system where we try to use the IR content with name authority records that we create here locally. We create authority records for all of the authors that contribute. We make use of GeoNames and its database for place names and geographic locations. We have a number of different subject lists we can use [such as] LCSH, MeSH, and FAST from OCLC as well as just keywords.… We make use of the extended date time format for time formats so you can go through and have a consistent machine-readable format for talking about dates.… Places, organizations, and people, we have a system for authority control on those as well.”

(s10).

In addition, person entity has a higher chance to be controlled by ORCID identifiers as some of the IRs are planning to adopt ORCID in their IRs:

“We were talking about ORCID. We were talking about pushing to get ORCID for every faculty member at the university. We are in discussion right now about serving ourselves into that space.”

(s1).

#### Tools

The interview data displays some of the current practices regarding tools for data curation in IRs. The tools include IR software, metadata schemas, ontologies, identifier schemas, controlled vocabularies, and applications for data curation S1 Table.

IRs use different software for their platforms (Table 7). Many of the IRs employed widely used solutions for repository services such as Bepress Digital Commons, DSpace, and Hydra. Digital Commons is software managed by an outside company called Bepress. IRs that use Digital Commons usually have fewer personnel available to work on software development. Some of the IRs, however, used local solutions for their specific goals and needs. IRs select their software based on available resources and needs. The performance and available personnel of an IR software development team affects the selection of IR software. Some institutions try to use the software that can be controlled and developed by them. Other institutions purchase services from an outside company to outsource the software management of their IRs. In addition, political factors as well as the software provider’s perceived reliability may affect decisions about IR software:

“I think that there were really only three players in the field in terms of platform. When they made the decision to go with DSpace, E-print was really only used by folks in the UK, so that was kind of off the table. DSpace has a lot of support and a big user community here in the US. Also, it [DSpace] was something that we could manage locally, and I think that's why we chose that over Bepress Digital Commons. Because our team, in the library technology unit, would always like to control the development and use of the software.”

(s2).

**Table 7:** Tools for research data curation.

**IR Software**	
Bepress Digital Commons, DSpace, Hydra, Dataverse, HUBzero, Aubrey, SobekCM	
**Metadata Schemas**	
Dublin Core (DC), Qualified DC, DataCite Metadata, MODS, METS, PREMIS, MIX, EAD	
**Metadata Schemas used in Supplementary Space**	
Darwin Core, EML, DDI, TEI, FGDC, ISO 19115 Geographical Metadata	
**Identifier Schemas**	
DOI, Handle, ARK, HTTP URI, Permanent local URL	
**Controlled Vocabularies**	
DC Contolled Vocabularies, Library of Congress Subject Headings (LCSH), Medical Subject Headings (MeSH), Faceted Application of Subject Terminology (FAST),Only with Hydra: DC RDF Ontology, FOAF, RDF Schema	
**Applications for Data Curation**	
Creating and Editing Metadata	Microsoft Word, Microsoft Excel, Text Editor (WordPad, Notepad++), Oxygen XML Editor, Morpho (Ecology Metadata Editor), Nesstar
Editing Images or Videos	SnagIt Photoshop for images, Handbreak for audiovisual
Cleaning Data	Open Refine
Storing Data	Dropbox, Google Drive
Identifying and Validating Data Files	DROID, PRONOM, Git for version control, FITS for file characterization
Transferring Data	BagIt
Indexing Data for Searches	Apache Solar
Tracking and Measuring Data	Altmetric

Some of the institutions have very specific purposes and needs for their IR platform. The system has to suit the other repository infrastructures within the institution, as well as all of the other local policies and norms for data curation:

“This is one that we have built locally.… It's a Python-based repository infrastructure we built.… then we have a sister piece, which is the archival storage repository that we use to build archival storage systems within our institution. Both of them are locally developed. We are in the process of sharing components of them, but we’re probably not going to be making them generally accessible because they're very, very tied to how we’ve wanted to do things.”

(s10).

The interview data indicate that most of the IRs provide simple descriptive metadata. Even though some of the IRs provide diverse metadata elements for specific disciplines, the elements that are regularly filled out by researchers are limited to required elements. Metadata for all of the IRs are based on the Dublin Core (DC) metadata schema or Metadata Object Description Schema (MODS), and some of the IRs offer additional metadata schemas such as DataCite metadata. One interviewee mentioned that they use DC metadata because it is simple for all researchers: “We have been using DC, because we want all researchers to be able to use it, so we keep it really simple.” (s3). Most of the IRs locally modified the DC metadata for their needs. Another interview participant stated that they use qualified DC metadata for everything in her IR as well as DataCite metadata for data objects:

“We allow people to put in qualified Dublin Core or DataCite [metadata], which they could do for either data objects or regular objects. If people have more specific metadata schemas, they need to add that as an attached file. The qualified DC is for everything, and then DataCite is optional.”

(s9).

The interview data also identified some metadata elements particularly added for research data. They include citation information, abstract, location, temporal coverage, methodology, note, collection, related URL, and partner institution. However, the main elements provided by most of the IRs are close to simple DC metadata elements [51]: title, creator, subject, description, publisher, contributor, date, type, format, identifier, source, language, relation, coverage, and rights.

In spite of the simple metadata elements from the IRs, research data is complex and diverse. Unlike most research articles, which are usually submitted in PDF format, data are submitted in different formats and types. The complexity and diversity of the data increases issues with creating and adding metadata, and then a lack of metadata is connected to issues with reusing, sharing, and searching the data:

“However, we sometimes add extra files. You can give us a survey; you may also give us a codebook; and you can also give us DDI file, or all three of them together. But it doesn't display out as metadata, and it's not really searchable in a structured way within DSpace.”

(s5).

Current practice for creating and adding metadata consists of filling out the form of metadata elements and creating and uploading separate files that include supplementary information about the data. Most of the IRs have adopted this practice of metadata creation. As mentioned earlier, the main metadata elements of the IRs are based on DC metadata, which is not sufficient to describe research data. The result of the insufficient metadata elements may result in abuse or repurposing of some the metadata elements:

“You have to give it [the data] a title, synopsis, and abstract. You could fairly abuse the abstract field. Near the end of the publication workflow, there is also a place you can add notes. That's a kind of space to catch all for the data.”

(s3).

The supplementary information cannot be searched in a structured way, but the information can be found by using full-text searches. Interview participants also mentioned some of the disciplinary metadata schemas currently used by researchers within their IRs for supplementary information. These included Text Encoding Initiative (TEI), Federal Geographic Data Committee (FGDC), Data Documentation Initiative (DDI), Darwin Core (DWC), Ecological Metadata Language (EML), and ISO 19115 Geospatial Metadata. In addition to the descriptive metadata, most of the IRs also support administrative, structural, and technical metadata (e.g., METS [Metadata Encoding and Transmission Standard], PREMIS [Preservation Metadata: Implementation Strategies]).

According to the interview data, IR software has its own identifier schema embedded into the established infrastructure. Many IRs employ the underlying identifier schemas in the software without a strong discussion about selecting an identifier schema that fits their specific goals. However, recent active movements toward the improvement of research data curation and semantic web technologies have changed the perceived importance of identifier schema selection for IRs:

“We use Handle because DSpace uses it. I think, in terms of the decision to move to DOI, it was made because DOI is becoming more and more important. And, there wasn't necessarily any sort of formalized process to choose that [DOI]. It was just kind of a recommendation from referred institutions.”

(s2).

Another interviewee also discussed her own ideas about her IR moving to or adopting a new identifier schema. The changes might not only satisfy the IR content providers but also expand the IR services:

“We do have researchers who are explicitly asking for DOI. We use Handle. Especially for publishers, I don't think they have the same knowledge of other identifier systems. They appear to be saying that you need DOI, so we are getting asked for DOI. Often when we probe a little bit about that [their need for DOI], we find out Handle is sufficient, but not preferred. So, researchers, at least in my experience, so far have been a little nervous about using something that is not DOI. That's not what they are familiar with in their publication process. I also think we are interested in potential citation tracking. We may possibly use DOI for that. In that way, I think there are two main reasons [to use DOI]: comfort level because of familiarity with DOI, and working with some of the services DataCite is trying to provide; as well as I understand, DataCite is working with CrossRef to provide more services for DOI.”

(s5).

For potential use in changing circumstances, the identifier schemas that the interview participants mentioned included DOI, ARK, Handle, HTTP URI, and permanent local URL (Table 7). Many of the IRs use the identifiers that came with their IR software, and if the IR staff identified a need to adopt or move to a new identifier schema, they were planning or testing the adoption of a new identifier. Most of the efforts came with the software that use Handle as a default. In addition to the IRs planning a change, some of the other IRs had already made changes. One interviewee discussed the identifier schema used in her IR. Her IR uses both Handle and DOI with DSpace software, but the use of DOI was optional. The researchers who want to be assigned a DOI for their data have to request one: “We automatically have Handles with the DSpace software, and then we also give an optional DOI, but it’s an opt-in. We don’t automatically assign them.” (s9). Another case was with Dataverse software. The repository also used both Handle and DOI, but only DOI was visible to the users of the repository. The interviewee strongly indicated that DOI was sufficient for his repository without Handle:

“Right now we just use DOI. Dataverse uses Handle as a default.…. We had to wait. Right now it [DOI] is appearing on the screen. We were trying to get them [Handle] to not show, but apparently it’s hardcoded in. We’re hoping they [Dataverse] are going to be coming up with a new version where I think we can remove the Handle and just have DOI.”

(s14).

A similar change also occurred with Bepress Digital Commons software, which does not use a currently existing persistent identifier. One of the interviewees said that his institution hires Bepress to manage his institution’s IR and operate DOI systems separately, in order to assign DOI to data. Hydra solution under Fedora Commons software not only provides a flexible identifier system environment but also supports a linked data integration platform. The interview data showed that the IRs using Hydra can employ various identifier schemas. They used DOI, ARK, or HTTP URI. One interviewee shared that her institution’s IR uses both DOI and ARK, and the two have different granularity levels for their assigned objects: “What I am seeing so far is that we assign DOIs at collection level, and ARKs get assigned to every single digital object within that collection” (s4). Another IR that uses Hydra also uses HTTP URI. In order to generate an identifier, the IR used software called Nice Opaque Identifier (NOID). NOID can generate two different types of identifiers for short- or long-term uses. The short-term identifiers are more like random namespace-less numbers, but the long-term identifiers are persistent object names like DOI, ARK, and Handle [52]. In the IR, researchers self-generate a NOID, and then the NOID is tacked on the IR’s URL, which develops a HTTP URI.

“The software is called NOID. … every file that’s uploaded gets one of these NOIDs, which is in effect a namespace-less verifiable unique identifier, and then what we do is we take that bare namespace-less identifier and we tack on our IR URL, so our approach to a persistent unique identifier is to use HTTP URIs that we mint in the IR.… As I just said, we do provide an identifier yield for every deposit. I don’t know how our users are using that field, so they could be self-populating DOIs or handles or other, maybe PubMed IDs.”

(s12).

Most of the IRs have not been using controlled vocabularies. However, two interviewees mentioned that they do have controlled subject lists, which contain Medical Subject Headings (MeSH), Library of Congress Subject Headings (LCSH), and Faceted Application of Subject Terminology (FAST). Two other interviewees also mentioned that they do not have any controlled vocabularies but use DC metadata type and certain controlled format list to organize the format or type of submitted contents.

One of the IRs is exceptionally different in its use of metadata. The IR uses Hydra solution under Fedora software. All of its metadata are modeled using RDF triples. The data is integrated with the concept of linking activity. Linked data principles [53] emphasize the use of HTTP URI. A datum is represented by a URI, and the two related URIs are linked by another URI. The three URIs accordingly form an RDF triple. The IR’s metadata for research data is a series of RDF triples. The IR uses locally modified DC metadata schema and sources of the elements from RDF ontology, Friend of a Friend (FOAF), and RDF Schema. One other interview participant indicated that her institution is planning to change IR software from DSpace to Hydra in order to fully set up an integrated linked data web.

The interview participants presented diverse applications or tools used within their data curation workflow (S1 Table). The applications are tied to the objectives of the activities supported by IRs: creating and editing metadata, editing images or videos, cleaning data, storing data, identifying and validating data files, transferring data, indexing data for searches, and tracking and measuring data (Table 7). In the process of editing metadata, different types of text, XML, or disciplinary metadata editors are used. Particularly, IRs widely use simple text editors to create ReadMe files that contain information about data files. Those files are normally used as supplementary metadata for data objects:

“Honestly, in many cases, we were actually creating [metadata on] just plain text files, so we do a lot of work with ReadMe files, we create just basic ReadMe files that have some amount of structured data. But often it is more of a big sort of place where researchers can talk in unstructured ways about the data. We get a huge variation in datasets. So, often there isn't a standard used. So, yes, for metadata, we tend to rely a lot on plain, readme.txt.”

(s5).

Another interviewee explained that the main tasks for him and his colleague are to make sure the data is understandable through the metadata documentation. In order to do that, the interviewee not only uses simple text file editors but also employs, in extreme cases, data and metadata conversion and editing tools (i.e., Nesstar). Nesstar is an advanced data management tool that helps the curators easily get DDI-formatted documentations. The interviewees also described conducting data cleaning for the researchers, but they only do it a little bit. The tool identified for the task was Open Refine, which helps clean data and transform data from one format into another.

Software tools or guidelines for data file identification and validation were another interesting finding identified from this interview data. IR staff use a file format identification tool (i.e., DROID) developed by the National Archives of the UK to perform automated batch identification of file formats, as well as a file format registry (i.e., PRONOM) to support digital preservation. They also used the GitHub repository for version control, due to the frequent events (e.g., updates) on data. File Information Tool Set (FITS) developed by Harvard University Library is also a useful tool for identifying, validating, and extracting technical metadata in file formats. IRs could use the file format metadata collected by the FITS for long-term preservation. To validate, transfer, and package data easily, IRs use BagIt, which is a tool developed by the Library of Congress and their partners in the National Digital Information Infrastructure and Preservation Program. The tool helped IR staff make OAIS Archival Information Packages in order to transmit the archival essence of data and its metadata into their IRs.

#### Policies, rules, and norms

The interview data analysis not only identified some current policies, rules, and norms that the IR staff use for data curation, but also the rationales for their actions around the practice. Some of the IRs currently are developing and improving their practices around policies, rules, and norms. Even though they have some established practices, they keep trying to figure out the best practices from various sources. One of the interview participants indicated that his institution provides data curation services through the IR, but the IR does not have many policies in place:

“We don’t have a whole lot of policies in place right now about what kinds of items get out, how they could be used, and how they're supposed to be done. Over the next year, we’re going to be looking at cleaning up some of these areas where we're having gaps in policies and documenting them fully in the hopes of doing a TRAC, Trustworthy Repositories Audit & Certification.”

(s10).

Some of the IRs tend to start their data service with minimal policies and rules, and then they develop and improve their policies and rules while they operate the services in a bottom-up approach. The sources of the policies they develop were also identified from the interview data. In many cases, the IR staff learn best practices from participating in various academic conferences and curation community training, taking coursework, and benchmarking peer institutions.

The policies identified from the interview data pertain to data management workflow, scope of data, deposit, copyright infringement, accessibility, collection, and preservation. In comparison to other policies, preservation policies have distinct differences between the institutions. One of the institutions has a fairly elaborate preservation policy. It has three different preservation levels, depending on the format of the material. If a material has a proprietary file format, or a file format that is not widely adopted, etc., the material would be categorized as low confidence level, which only provides basic preservation. If a material satisfies the criteria for moderate confidence level or highest confidence level, the material would be preserved by the corresponding level’s preservation actions: “We have some standard preservation activities that are running. I will say that we have different preservation levels depending on the format of the material.” (s5). A few IRs also have preservation contracts with the data providers storing datasets. When a dataset is submitted into these IRs, it automatically has a set-year contract (e.g., 5 years or 10 years). When the initial contracts are terminated, the IR staff and the data providers evaluate the datasets for long-term preservation. The duration of the contracts are different between the IRs. One institution used a 5-year model, and the other used a 10-year model. There is not an agreed-upon model for the contract; they seemed to select the period based on their experiences or other policies existing in their institution:

“Our institution has a policy. It’s one of the few schools that ever got their act together and has a data retention policy, where they require 5 years retention of data for any publication; that’s where we got our 5 years. I think NFS and other funders vary as far as how long they ask for data to be shared; it could be 3 years or unspecified. I think we mostly got 5 years from our institution policy. It seems like actually we don’t really know how long data stays useful, so we figured 5 years is a good starting point, and then we’ll see how that goes. Some researchers have said, ‘We think it should be forever.’”

(s14).

There are some recommendations for data curation services [54,55], including metadata fields for research data and recommended file formats. The IRs could make them requirements. However, they tend to make them be recommended guidelines in order to keep a higher volume of data in their IRs. They try to keep fewer policies, but then have recommended guidelines: “We do have format guidelines. I am sure you know that people aren't running to put stuff in most institutional repositories, so you really don't want to constrain them.” (s15). One other interviewee also mentioned loose guidelines rather than strict policies. The IRs also use data curation service guidelines (e.g., Data Curation Profiles Toolkit, Data Management Plan (DMP) Tool) developed by well-known institutions. However, many of the IRs used the guidelines with local modifications. They tended to tailor the guidelines for their needs:

“Data Curation Profiles Toolkit, you know, we have modified that when we meet with the researcher, because a data curation profile isn't essentially a data-oriented reference interview. It is just extremely detailed.… So, we do stick within those concepts, but for the most part, it's really slimmed down.”

(s15).

There is a norm identified by the interview data: some of the IRs apply a subject specialist-centered service norm in their curation services. Because of the complex and diverse types of research data, the roles of subject specialists who can provide support based in disciplinary knowledge and practice are emphasized within the data curation processes in IRs:

“We always recommend that people use norms from their disciplinary practice. We don’t have a full list of those written, but we refer to them. That’s one of the reasons that we work through this specialist or liaison model to make sure that we do know those norms from the different communities for any of the technical data support that we’re doing.”

(s11).

#### Contradictions

In an activity system, contradictions can be understood as tensions, conflicts, or limitations among the components of that system [44,56]. Identifying the contradictions that exist in and between activity components and seeking to resolve those contradictions can lead to the evolution of and innovation in the activity system [44]. This section provides different examples of contradictions that occur between components of an activity structure, and some solutions for resolving those contradictions are suggested. A better understanding of the types of contradictions found in IR curation work can benefit the institutions that currently plan to implement institutional data repositories. In addition, some alternative solutions for the identified contradictions are suggested to help evolve the activity systems.

There is a tension closely related to the issue of tradeoffs. One institution only uses a Handle identifier system because of limited resources in its IR infrastructure. In order to adopt a new identification system, the institution had to consider the tradeoff between tool complexity and scalability. They seemed a little afraid of adopting a new system:

“Definitely, part of the reason we only use Handles is we don't have a generator to create a unique identifier. Because as soon as you start doing that, you will get into a lot of identifier criteria and quality problems. You will not be able to foresee it. If you come up with a simple system, then it's not going to be scalable, but if you come up with something that's really scalable, it's probably going to be not very simple.”

(s15).

One of the interviewees provided an example of a contradiction that occurred between a dataset and his IR software. The contradiction occurred during the process that satisfies the curation objective of data storage or preservation. Research data is very complex and diverse, and the number and the scale of data files and their types are also very different depending on the domain of the research. However, based on the interview data, the existing IR software and storage space did not yet sufficiently support the needs of research data curation services in IRs. An interviewee mentioned a workaround [48] to avoid the contradiction. His IR uses a mirror repository as a backup system, a form of workaround, which is used for large research datasets. The mirror stores data files and then it has a link to the data description in the IR.

Another interviewee provided a different example of a contradiction between objective and tool. In response to the interviewer’s question about the levels of identifier granularity for supporting identification of entity level data (i.e., What identifiers do you use at the data collection/set level, file/object level, or entity level?), she simply said, “That’s a system limitation.” (s9). Granularity is the extent to which the identifier system allows data to be referenced at a different granularity [43]. She explained that her IR does not assign data identifier strings to entity level objects, because her IR software does not support the service. Throughout the interview data, many interviewees indicated that their data curation services frequently depend on what can be supported by their IR software. To resolve this contradiction, IRs can adopt an additional and external identifier system that can be assigned to an entity level’s objects. Also, IRs can replace their existing IR software with one that supports linked data/RDF technology. With such software, diverse existing controlled vocabularies that can be used to construct HTTP URIs can be assigned to the entity level’s objects.

Analysis of the data identified contradictions that exist between tools and best practices. One of the interviewees indicated that his institution might adopt a new identifier system for the person entity to provide an effective authority control service, but the progress on it is very slow. One of the main reasons for the slow process is a lack of established best practices. Knowledge about application of the new system was insufficient to actually adopt the system with low risk in his IR. His institution discussed the system adoption, but progress moved forward slowly without examples of best practices:

“The ORCID ID, I would love to see more uptakes. I think maybe one percent of the people are really excited about it and using it. Ninety-nine percent of the people know that's out there and just think it's one more thing.… I think a lot of them are primarily concerned with getting it into the systems that they use. So, I don't know. We were watching it. We definitely want to implement it here. We've talked about adding either just dumb flat text ORCID field to DSpace or maybe one that was actually connected by the API to ORCID services. We just haven't made much progress on it. We are watching to see what’s evolved.”

(s15).

To resolve this type of contradiction, IRs can conduct a pilot project to test the system performance. One of the interview participants mentioned her IR’s pilot test for using DataCite DOI. She also explained that her institution would decide whether to adopt DataCite DOI or not based on the test result:

“We are just initiating a pilot to use the DataCite DOI for research data. We haven't started to do that yet. We have a handful of pilot participants we are going to contact. Essentially, we would be working with them to submit the item into our repository so we would get a handle, but we also find a DataCite DOI for the item, research data.”

(s5).

In the context of IRs, many different resources affect the curation services or tools of IRs [8,13]. The interview data also identified various resources intertwined with the services or tools. Some of the interviewees implied that the current availability of personnel affects their usage of tools. Some IRs control and maintain their IR server and systems with a strong development team; otherwise, if they don’t have a strong development team, IRs may outsource the management of their IR server and systems. One of the interviewees stated that her IR has a strong development team:

“Our team in the library technology unit always likes to control the development and use of software.… Mainly we issued the resources in terms of people we had around to do the data modeling and then think throughout about the issue and the programmer power we have available to enact a solution.”

(s2).

Financial funding is another resource affecting IR services. The annual budget for IRs is limited, and variations in budget size influences the kinds of services and tools that the IRs provide: “We need to consider the incremental cost for support services so we see how much they actually cost. We haven’t even figured out what extended storage costs will be.” (s14). As mentioned in the previous sections, the current tools and available best practices are also resources that impact IR data curation services.

Some of the contradictions recognized in the interview data involve components of data curation work in IRs. One interview participant presented a contradiction that occurs between norms, best practices, and division of labor. Her IR adopted a new model for identifier granularity, but the IR platform did not actually employ the new model. Her IR team did not have access to sufficient resources to use the model in their setting. For example, they did not have norms or best practices to help them adapt the new model to their existing IR setting. Also, her IR has insufficient human resources to devote to the software development that would have been necessary to implement the new model:

“We looked at and adopted the Dryad model for [identifier] granularity. … [However] We didn't see a model for how we would make it work in our IR platform [DSpace]. Mainly we had issues with resources in terms of the number of people we had around to do the data modeling and think through issues and the programmer power we have to enact. We didn't have either of those resources. So, we just decided on very cut and dry DOI issuing.”

(s2).

One last example of contradictions identified from the interview data occurred between the IR infrastructure and researcher needs imposed by publishers. Researchers want to follow the practices recommended by publishers, and therefore ask their IRs to meet those requirements. However, IR infrastructure is not always aligned with the publishers’ practices:

“We do have researchers who are explicitly asking for DOI. We use Handle. Especially for publishers, I don't think they have the same knowledge of other identifier systems. They appear to be saying that you need DOI, so we are getting asked for DOI. Often when we probe a little bit about that [their need for DOI], we find out Handle is sufficient, but not preferred. So, researchers, at least in my experience, so far have been a little nervous about using something that is not DOI. That's not what they’re familiar with in their publication process.”

(s5).

One solution for this contradiction could be designing a pilot test to determine the value of adopting suggested tools or instruments. Based on the test result, IRs would be able to make an informed decision about the adoption.

### IR staff role-related skillsets

The interviewees discussed skillsets that they think they need for research data curation. The identified skillsets can help develop a team of data curators’ professional expertise. Most of the skillsets discussed are interchangeable between the roles of IR staff, but we mapped the skillsets based on the staff’s role-related activities (Table 8). Cells shaded grey mean that the skillset is more or less limited to the specified role. The main skillsets contain eight distinct types of knowledge or skills, including metadata, domain knowledge, research practice, curation lifecycle, software, library technology skill, data description and documentation, and communication. Almost all of the interviewees mentioned knowing how to create metadata as a necessary skill. One of the interviewees clearly expressed that hiring someone who already knows metadata and diverse disciplinary practices will be more efficient in completing their work:

“As far as the training for the folks working with the creation of metadata… That [the training] is usually where the most work has to be done when bringing in someone new. Because it just takes a while to see all the different variants that you have. You can have documentation, but it takes a lot of time. You just need to see a variety of different kinds of content work with a variety of different disciplines to understand how they differ.”

(s10).

**Table 8:** IR staff’s role-related skillsets.

Head	Data Curator	IR Manager	Metadata Specialist	Developer	Subject Specialist	Graduate Assistant
Understanding of data curation lifecycle						
	Long term preservation of knowledge					
	Familiarity with research data (e.g., Ability to handle data complexity and diversity)	Collection management skill	Metadata knowledge particularly for research data	Technical details of repository software, server, and its architecture	Understanding disciplinary metadata, workflows, and knowledge	
	Academic research practice	Software skill			Collection management skill	
Library practices, needs, and technologies; Ability to communicate and work within a team; Data management practice; Data description/documentation skill; Soft skill (i.e., communication); Time management						

Another interviewee considered knowledge of metadata and experience in creating metadata as an important skill for IR staff:

“My theoretical conception of what metadata is and what it should be doesn't align very well with what the researcher’s conception is. There has to be some kind of compromise there, and we try to understand how we take this. There are some highly detailed and highly formalized schemas, and then take these things [data] that are really amorphous, and how do you put those things together, and still have metadata that is useful and serves its purposes? I think that is going to be one of the technical skills that we are going to have more of.”

(s1).

An interviewee even mentioned metadata knowledge particularly for research data as the most important and ideal skill for staff who work on research data curation:

“The most difficult thing that we struggle with is the metadata on all three of those levels [data curators, data providers, and users]. That would be the ideal skill for someone to have, to be someone who can figure out the correct level of metadata and the range of data.”

(s9).

In order to support metadata creation, IR staff need to understand disciplinary knowledge, research practices, and the data curation lifecycle (workflow). One interviewee emphasized domain knowledge as an important skill for data curators: “Domain knowledge, especially data complexity and diversity to bridge metadata librarians and researchers.” (s4). Another interviewee highlighted the importance of understanding academic research practices:

“Obviously, familiarity, deep familiarity with research practices in academic research and its research data needs, experience with and knowledge of library systems and practices, and how each of those operate with the other.”

(s11).

However, this does not mean the IR staff need to be biologists, chemists, or physicists. One other interviewee stated that his institution does not hire librarians with degrees in fields other than library studies. Instead, his team wants people who know the data curation lifecycle and its workflow:

“Librarians who are willing to understand some of the various research methods that get used within these disciplines, and are able to communicate and ask questions to learn more about those datasets and different kinds of scholarship. I don’t need librarians with degrees in Biology to go talk to a biologist. I want people that know what we’re going to do with the data once we get it and to be able to ask questions of the biologists that get them to give more meaningful information.”

(s10).

Managing software and understanding library information technology is another skill or knowledge needed for IR staff. However, IR staff have different levels of familiarity with certain technologies. One interviewee tried to explain what the right amount of technological knowledge might be:

“I would say it is substantially different in that it depends on what the data is. We had to use tools you know and understand. [For example,] Excel. There is a difference in how to use Excel and how to tease or parse data in Excel. Also, using an Excel spreadsheet for multiple sheets—what that means and the problems with that.… In order to have them [IR staff] ask the right questions… in my mind, it's little more than just having a certain level of comfort working with software.”

(s5).

An interviewee introduced a typical case illustrating the need for technological skill:

“We prefer to provide a text file because it is more accessible to people, and then we work with them to create a text file version of that [original data file]. Some researchers are comfortable with that; they say, “Okay, I'll just export it and give you another version.” Some people say, “I don't know how to do that.” So, we have to handle the process.”

(s1).

The final skillsets identified for IR staff include interpersonal communication and documentation skills. Some of the interviewees stated that interpersonal communication skills are important because research data curation is a relatively new field, which means there is a lot of uncertainty or ambiguity. Clear communication between IR staff and data providers enable smooth data curation processes:

“Communication, I would say it's even more important because it's a relatively new part of the field. There's a lot of uncertainty, there's a lot of ambiguity, the researchers are not always comfortable with talking to somebody who might not be a domain expert about the data and managing their data, and so just being able to have an intelligent and productive conversation with people is far more important than whether you can write the script of Python or whatever.”

(s15).

One other interviewee also explained that both communication and documentation skills are important for increasing researchers’ trust in their services and encouraging them to share their data. One of them stated:

“The research data, that’s really a big deal and you’re asking them to give it to you? This is either more work for them, or you’re asking them to trust you with walking their baby. They really need to feel comfortable and to feel supported. They need to know that you know the answers or that you’re going to find out the answers. A huge amount of work goes into establishing and supporting trust. Some of that trust is with documentation.”

(s11).

Another group of the interviewees described IR staff as almost all soft-skilled people. In order to complete all of the research data curation processes, a significant amount of presentation, communication, documentation, and teamwork skills are required in IR staff. One of them described the tasks that need soft skills:

“Our data curators are almost all soft-skilled people. They go to promote our services, and they work with a lot of faculty members on their data management plans. They are using the right language and explaining things like the difference between backup and preservation.”

(s9).

## Discussion

### Activities in the IRs

According to Activity Theory [44,45], activity can be generally understood as interactions between a subject and an object in a community, and the activity is mediated by contexts that include tools/instruments, policies, rules, norms, and division of labor. Various activities and their mediating factors exist in the context of IRs; identifying them and investigating their relationships can form a useful knowledge base that can be used in data curation planning and education, as well as in designing and implementing research data services in IRs. Data activities in the IRs could mainly be divided into two different categories: curation activities and other related activities (Table 1). Curation activities include the activities that are directly related to data curation work; on the other hand, other related activities contain the activities associated with administration/management and user services of the IRs. Collecting knowledge about those identified data activities can guide institutions that currently provide or plan to provide institutional data repository services. In addition, understanding those data activities provides insights for further developing a general model of IR research data curation work.

There are many different general models of research data and related curation activities (DCC Curation Lifecycle Model, DataOne, OAIS). The curation activities identified in this study can be mapped to the DCC Curation Lifecycle Model. The mapping demonstrates the differences and similarities between a general model and the IR data activities identified in this study; understanding these similarities and differences can then generate further discussions among members of research data curation communities regarding the nature of curation activities within the IR context. The DCC model provides an overview of the stages required for curation of data from conceptualization and receipt through the publication and sharing of data [31]. The IR curation activities appeared throughout all of the sequential actions of the DCC model, and identified relations with the full lifecycle actions of the DCC model (Table 9). The IR data curation activities were more detailed than the sequential actions of the DCC model. For example, *consulting with researchers*, *communicating with IR or library staff*, *developing and documenting metadata*, *validating data*, and *packaging dataset* can be mapped into the *preservation action* stage of the DCC model. In the *preservation action* stage, curators communicate with researchers to develop and document metadata for their specific research data. Within that communication process, IR or library staff who have domain knowledge in the research discipline may be involved in the process of developing metadata. After the process, curators and researchers collaborate on a validity check of the data and then package the data in order to store it within the IR. In addition, one particular IR action (i.e., *consulting with researchers*) coincided at least with one other action via all of the sequential actions. The *consulting with researchers* action is a full lifecycle action, and its coincidence with different actions demonstrate the importance of communication and consultation between curators and researchers. In some cases of data curation services, curators are involved in the research projects from the planning stage, and the involvement is continued until the project ends. Some of the interview participants mentioned their wide spectrum of different points of involvement in data curation. The curation work can include meetings with researchers who are interested in storing and preserving data; providing assistance in developing grant proposals; regularly meeting with researchers to help them deposit their data throughout their project period; and helping transfer data from one place to another. In order to understand data practices (e.g., objectives, division of labor, data types, tools, rules, norms, policies) of a specific research community and to curate the data in the ways that aligned with those practices, communication between IR curators and data providers throughout the curation lifecycle is essential.

**Table 9:** The comparison of the IR curation activities to the DCC Curation Lifecycle Model.

	**DCC Full Lifecycle Actions**							
	Description and representation of information							
	Preservation planning							
	Community watch & participation							
	Curate and preserve							
	**DCC Sequential Actions**							
	Conceptualize	Create or receive	Appraise and select	Ingest	Preservation action	Store	Access, use, and reuse	Transform
**IR Curation Activities**								
Understanding data curation needs								
Interviewing researchers	x							
Consulting with researchers	x	x	x	x	x	x	x	x
Communicating with IR or library staff			x		x			
Managing and sharing data								
Receiving or transferring data files		x						
Cleaning data			x					
Converting data to a different file format				x				
Developing and adding metadata					x			
Validating data					x			
Packaging data				x	x			
Uploading and publishing data into IR						x		
Ensuring that data is accessible and reusable								
Annotating data for relevant entities							x	x
Optimizing data to search engine							x	
Keeping data up to date in mirror repository						x	x	x
Re-evaluating data for long term preservation								
Selecting dataset for long term preservation							x	x

### IR activity structure

**Fig 1:**
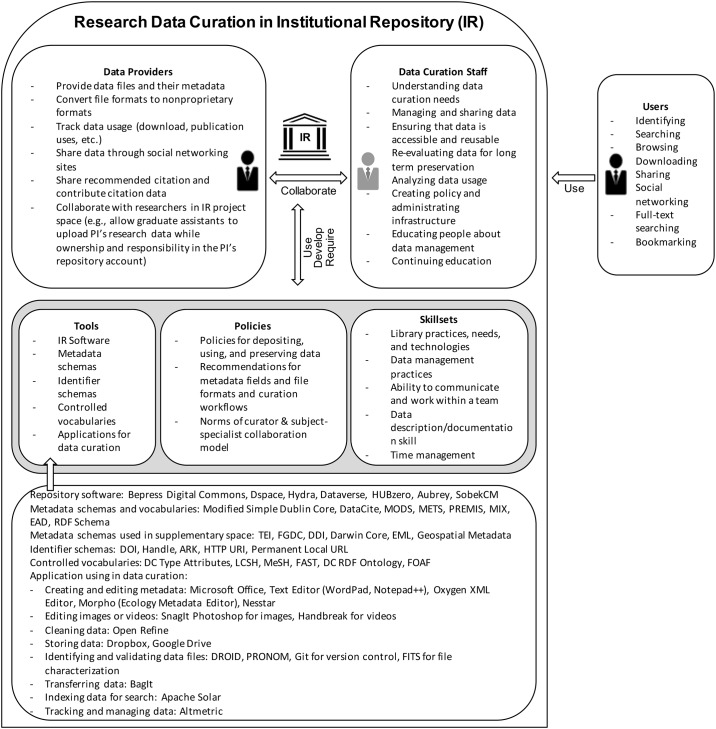
The structure of data curation in IRs.

The interview data identified seven different IR staff roles that provide research data curation services in IRs and the roles of data providers and data users. The roles of IR staff are head, data curator, IR manager, metadata specialist, developer, subject specialist, and graduate assistant. In practice, role mapping to job title is not consistent. Institutions may use different job titles and there could be many relationships between the data curation roles and the job titles based on local needs (Table 4). In addition, since IR staff collaborate, their tasks and required or preferred skills often overlap (Table 8). However, each role’s unique tasks and skillsets were also identified by the interview analysis (Tables 5 and 8). The knowledge of IR role-specific curation tasks and skills needed can benefit the institutions planning to implement data curation services and to recruit new members for their IR data curation teams. Furthermore, a design chart of research data curation in IRs including different data activities and their contexts (i.e., tools, policies, skillsets, division of labor) can provide ideas for how to implement data services in IRs (Fig 1).

Investigating major types of research data and their entity types has significant value for understanding different characteristics of research data [38] and for enabling the semantic data linking envisioned by Berners-Lee [57]. Research data in the IR context includes any type of data (e.g., text documents, spreadsheets, slides, audio recordings, audio-visuals, images, laboratory notes, statistical data files, databases, software codes, executable files, and tabular data files). According to the interview analysis, all of the IRs do not have any restrictions on the types of research data they will receive, but do have minimum criteria for acceptable data characteristics (e.g., file capacity, the number of files, and proprietary file extension). The minimum criteria and IRs’ discipline-independent nature enables the IRs to include all types of data from any discipline. For example, the IRs contain a greater variety of data types (i.e., raw data, text documents, slides, laboratory notes, spreadsheets, software codes, drawings, statistical data files, Website, and databases) than the data types of a Condensed Matter Physics community [29].

### IR staff skillset

**Fig 2:**
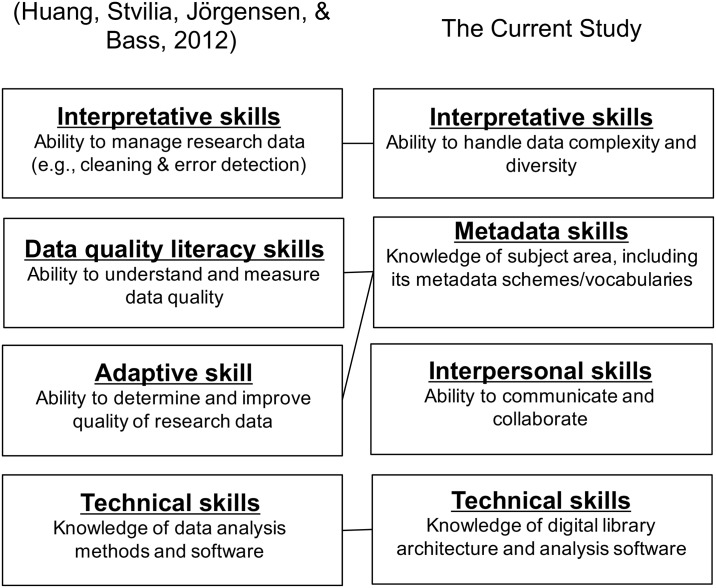
Comparison of IR data curation staff’s skills to data curation skills identified by Huang et al. (2012).

Comparing the IR staff skills identified by this study to the set of data skills needed for genome annotation curation (which is a type of data curation) identified by Huang et al. [58] reveal some differences and similarities (Fig 2). Interpersonal skills to communicate and collaborate with researchers do not have a clear match in the Huang et al. model. However, similar skills in his model fall under adaptive skills, which are the skills needed to determine and improve quality (e.g., value and relevancy) of research data. The current study’s metadata skills, which include disciplinary knowledge and its associated metadata knowledge, can be mapped to data quality literacy skills (which are the skills needed to understand and measure data quality) and adaptive skills. However both skills from Huang et al. contain many more detailed concepts than the metadata skills from the current study. For example, data-quality dimension, data-quality measurement, data-quality implication, data-quality cost/benefit, data-entry improvement, change process, organization policy, user requirement, and information overload are detailed concepts of the two skill constructs. Some of the differences in the number of detailed concepts between the current study and Huang et al.’s could be linked to the variations in the levels of data curation provided by IRs and subject-specific data repositories. Subject-specific data repositories and their staff are expected to provide deeper analysis of submitted data, including data annotation and quality assessment. Hence, curators of subject-specific data repositories are expected to be subject specialists with advanced degrees in those subject areas. On the other hand, curators in IRs collaborate with subject specialists to complete their tasks in research data curation. Similarly, Wu [59] identified 16 different data curation skills in the context of biological ontology. Based on her interview data collected from biocurators and Gene Ontology users, domain knowledge (i.e., basic biological knowledge, domain-specific biological knowledge, staying current on developments in biological knowledge, reading scientific literature, bioinformatics) was the most frequently mentioned skill for data curation. Besides domain knowledge, interpersonal skills, interpretative skills, and technical skills were also identified as data curation skills in biology.

## Conclusion

This study examined research data curation practices in IRs based on Activity Theory [44,45]. The study identified activities performed by IR staff, the activity context, and role-specific sets of the activities and skillsets in IRs. Based on the findings and discussion, we provide the following recommendations or curation knowledge that can benefit institutions that currently manage or plan to implement institutional data repositories.

### Designing effective teams for IR data curation

IR staff first communicate with data providers to understand the providers’ research data. This helps the staff coordinate the rest of the curation-related activities with skilled personnel. During the coordination process, communication among IR staff frequently occur to tailor the curation activities to the data provider’s needs and the types of data submitted. Once the coordination is completed, each activity and their actions are conducted throughout the data and data curation lifecycles. Although the activity of *understanding data curation needs* mainly requires communication between stakeholders, the rest of the activities also require a fair amount of communication. Research data curation is a comprehensive service throughout a data lifecycle; data curators must continuously plan, collect, assure, describe, preserve, discover, integrate, and analyze data [25,60]. Communication between the curation team and data providers throughout the data lifecycle is an essential task. One of the interview participants mentioned that he, as a data curator, spends about 80% of his time on communication. Because of the importance of communication for successful data activities, IR staff consider an individual’s communicative skill when designing an effective team for research data curation. In addition, data curation teams prefer having IR staff with research experience who also know data and data curation lifecycles. The identified division of labor among curation staff indicated the importance of networks and relationships between IR staff and domain experts (e.g., subject specialists/librarians, departmental collection administrators) in order to create and add metadata for different research datasets. Having the skills needed to develop software within a data curation team makes it easier to maintain the IR system. In the instances when an IR team does not have such skills and have to rely on the expertise of a different team within the same library, keeping a good relationship between the IR data curation team and the other team can still render an effective solution for data curation.

### IR infrastructure for research data curation

Research data tends to be complex and diverse. All of the IRs that participated in the study do not have restrictions on the types of research data they will receive. Only some technical limitations, such as file scale and the number of files, exist. In contrast with the diversity of accepted research data types, the controlled entity types used to identify complex research data do not reflect their complexity. The major controlled entity types used by the IRs are not different from the entity types of general library objects (e.g., books, journal articles). Instead, the IRs use supplementary ReadMe files to add domain specific metadata for the datasets. Although the supplementary information is indexed by the search engine(s) used in IRs, the information is not as effectively and efficiently searchable as the controlled entity metadata. This suggests directions for future studies, including future studies of scientific metadata and data identifiers. More specifically, it is important to determine what an optimal set of metadata elements is for research data and what current data identification schemes can be used to reference and/or identify different data entities.

IR staff use diverse tools/instruments for different activities and purposes. Although many of the tools are designed for similar purposes, they have slightly different goals and uses. IR software is a typical example. All of them support general repository functions (e.g., storing, publishing, sharing), but they have different characteristics in their administration, data storage and service components. Institutions should consider a variety of factors, such as the data curation needs of target communities, policies, norms, rules, and division of labor when they select tools for their institutional needs. In addition, institutions should think of future functionality needs. Many of those tools do not have sufficient expandability for their functions.

Policies, rules, and norms enable the IR system and the curation teams to be systematic in their practices, but the research data curation field is still an emerging community. As a result, the current practices of the IRs that participated in the study are not yet unified or standardized among themselves. Policies, rules, norms, and current practices were identified in this study. The institutions that currently manage institutional data repositories can refer to the current data curation practices, and they can also share knowledge about their own practices to the community.

When an institution develops its IR, the institution needs to consider the user services that can be provided through the IR. Without active IR content-contributors and end-users, it would be difficult to justify the cost of IR establishment and upkeep. Thus, providing appealing user services is important. Designing IR user services that can increase institutional members’ IR use and enhance IR staff’s outreach services is an activity to consider before IR implementation. All of the IR staff that participated in the study mentioned the outreach services they provide in order to increase the use of their IRs. The interview data identified different IR user services that are currently supported by the IRs. The IRs currently provide typical repository services (i.e., identifying, searching, browsing, and downloading) and several optional services (i.e., sharing, social networking, full-text searching, and bookmarking).

IRs operate in the sociotechnical context (i.e., tools/instruments, policies, rules, norms, culture, and division of labor) of their community. There could be conflicts and contradictions among different components of that context. For example, some tools cannot be used without using another tool. A tool cannot be used because of limitations in other resources (e.g., availability of software development expertise, budget, and policy). In order to avoid and/or resolve those contradictions, institutions must set a distinct goal and a plan for designing and implementing their IR systems, and those plans should attend to the various sociotechnical factors that may affect the IR’s effectiveness.

The findings of this study can inform the development of best practices, infrastructure configuration templates, as well as education in research data curation in LIS schools. The findings also indicate future research directions, such as: exploring data curation practices of IRs that use a specific tool; investigating IR user services to find ways to better motivate researchers’ IR use, and to design and manage IR user community(s). In addition, similar studies that examine goals, perceptions, and uses of IRs from the perspectives of data providers, end-users, or university administrators can help the IR community overcome various challenges associated with the operations of IRs. Finally, a future study exploring existing institutional barriers to establishing IRs at universities that do not have them yet would be of great benefit to the community.

## Supporting information

S1 Table Tools. (DOCX)
