# Supplementary material for: Practices of research data curation in institutional repositories: A qualitative view from repository staff
Source: PLoS One. 2017 Mar 16;12(3):e0173987. doi: 10.1371/journal.pone.0173987 (PMC5354423; doi:10.1371/journal.pone.0173987)
Supplement: S1 Table — (DOCX) [file pone.0173987.s001.docx]

# S1 Table. Tools.

| **Categories** | **Tools** | **URLs** |
| --- | --- | --- |
| IR Software | Bepress Digital Commons | http://digitalcommons.bepress.com/ |
|  | DSpace | http://www.dspace.org/ |
|  | Hydra | http://projecthydra.org/ |
|  | Dataverse | http://dataverse.org/ |
|  | HUBzero | https://hubzero.org/ |
|  | Aubrey | Locally developed software |
|  | SobekCM | http://ufdc.ufl.edu/sobekcm |
| Metadata Schemas | Dublin Core | http://dublincore.org/ |
|  | DataCite | https://schema.datacite.org/ |
|  | MODS | http://www.loc.gov/standards/mods/ |
|  | METS | http://www.loc.gov/standards/mets/ |
|  | PREMIS | http://www.loc.gov/standards/premis/ |
|  | MIX | http://www.loc.gov/standards/mix/ |
|  | EAD | http://www.loc.gov/ead/ |
|  | TEI | http://www.tei-c.org/index.xml |
|  | FGDC | https://www.fgdc.gov/metadata/geospatial-metadata-standards |
|  | DDI | http://www.ddialliance.org/ |
|  | Darwin Core | http://rs.tdwg.org/dwc/ |
|  | EML | https://knb.ecoinformatics.org/#external//emlparser/docs/index.html |
|  | ISO 19115 Geographical Metadata | http://www.iso.org/iso/catalogue_detail.htm?csnumber=26020 |
| Identifier Schemas | DOI | http://www.doi.org/ |
|  | Handle | http://www.handle.net/ |
|  | ARK | https://wiki.ucop.edu/display/Curation/ARK |
|  | HTTP URI | http://www.w3.org/DesignIssues/HTTP-URI.html |
| Controlled Vocabularies | LCSH | http://id.loc.gov/authorities/subjects.html |
|  | MeSH | https://www.nlm.nih.gov/pubs/factsheets/mesh.html |
|  | FAST | http://www.oclc.org/research/themes/data-science/fast.html?urlm=168918 |
|  | RDF Ontology | http://semanticweb.org/wiki/Ontology |
|  | FOAF | http://xmlns.com/foaf/spec/ |
|  | RDF Schema | http://www.w3.org/TR/rdf-schema/ |
| Applications for Data Curation | Microsoft Office | https://products.office.com/en-US/ |
|  | WordPad | http://windows.microsoft.com/en-us/windows7/products/features/wordpad |
|  | Notepad++ | https://notepad-plus-plus.org/ |
|  | Oxygen XML Editor | http://www.oxygenxml.com/ |
|  | Morpho | http://knb.ecoinformatics.org/morphoportal.jsp |
|  | Nesstar | http://www.nesstar.com/ |
|  | SnagIt | https://www.techsmith.com/snagit.html |
|  | Handbreak | https://handbrake.fr/ |
|  | Open Refine | http://openrefine.org/ |
|  | Dropbox | https://www.dropbox.com/ |
|  | Google Drive | https://www.google.com/drive/ |
|  | DROID | http://www.nationalarchives.gov.uk/information-management/manage-information/policy-process/digital-continuity/file-profiling-tool-droid/ |
|  | PRONOM | http://apps.nationalarchives.gov.uk/PRONOM/Default.aspx |
|  | Git | https://git-scm.com/ |
|  | FITS | http://projects.iq.harvard.edu/files/fits/files/fits_poster_final.pdf |
|  | BagIt | https://wiki.ucop.edu/display/Curation/BagIt |
|  | Apache Solar | http://lucene.apache.org/solr/ |
|  | Altmetric | http://altmetrics.org/manifesto/ |
